# One versus Many: Polymicrobial Communities and the Cystic Fibrosis Airway

**DOI:** 10.1128/mBio.00006-21

**Published:** 2021-03-16

**Authors:** Fabrice Jean-Pierre, Arsh Vyas, Thomas H. Hampton, Michael A. Henson, George A. O’Toole

**Affiliations:** a Department of Microbiology and Immunology, Geisel School of Medicine at Dartmouth, Hanover, New Hampshire, USA; b Department of Chemical Engineering, University of Massachusetts, Amherst, Massachusetts, USA; c Institute for Applied Life Sciences, University of Massachusetts, Amherst, Massachusetts, USA; University of Pittsburgh School of Medicine

**Keywords:** cystic fibrosis, microbial communities, antibiotics, biofilms, metabolic modeling, microbiome, chronic infection, cystic fibrosis

## Abstract

Culture-independent studies have revealed that chronic lung infections in persons with cystic fibrosis (pwCF) are rarely limited to one microbial species. Interactions among bacterial members of these polymicrobial communities in the airways of pwCF have been reported to modulate clinically relevant phenotypes. Furthermore, it is clear that a single polymicrobial community in the context of CF airway infections cannot explain the diversity of clinical outcomes. While large 16S rRNA gene-based studies have allowed us to gain insight into the microbial composition and predicted functional capacities of communities found in the CF lung, here we argue that *in silico* approaches can help build clinically relevant *in vitro* models of polymicrobial communities that can in turn be used to experimentally test and validate computationally generated hypotheses. Furthermore, we posit that combining computational and experimental approaches will enhance our understanding of mechanisms that drive microbial community function and identify new therapeutics to target polymicrobial infections.

## Abstract (precis)

Culture-independent studies have revealed that chronic lung infections in persons with cystic fibrosis (pwCF) are rarely limited to one microbial species. Interactions among bacterial members of these polymicrobial communities in the airways of pwCF have been reported to modulate clinically relevant phenotypes.

## PERSPECTIVE

Cystic fibrosis (CF) lung disease results from a cascade of events that begin with mutations in a single gene coding for an ion channel, *CFTR*, leading to defective chloride secretion and reduced airway surface liquid hydration (1). Loss of CF transmembrane conductance regulator (CFTR) function impairs mucociliary clearance, creating an ideal environment for pathogens, leading to chronic infection, inflammation, lung damage, and increased rates of mortality (2). As described below, emerging evidence from *in vitro* coculture systems and animal models is beginning to identify how the polymicrobial nature of chronic airway infections affects health outcomes of persons with CF (pwCF).

Multiple reports highlight that prevalent and abundant bacteria found in the CF lung, including Pseudomonas aeruginosa, Staphylococcus aureus, *Streptococcus* spp., and anaerobic species, are capable of engaging in multiple interactions impacting clinically relevant features such as virulence, persistent colonization of the airways, and antimicrobial recalcitrance (3–8). More specifically, P. aeruginosa and S. aureus, two of the most studied CF lung pathogens, have been shown to (i) negatively affect respiratory function of pwCF when they cocolonize the airways compared to when they are present alone (9–11) and (ii) shift antimicrobial efficacy (including the front-line CF drug vancomycin) through metabolic interactions (12–15). Other groups have used coculture and mouse models to understand underlying mechanisms of how microbial interactions can affect clinical outcomes for pwCF (16–18).

While these above-mentioned studies support the concept that the relationship between microbial interactions and clinical outcomes is complex, the pathogenesis of infections observed in CF lung disease is still poorly understood. However, is it possible to define a community (or communities) that would explain the consequences of polymicrobial interactions in the context of the CF airway, as well as the impact of community interactions on antibiotic responsiveness and disease outcomes? Having such model communities could provide tremendous benefits in terms of (i) focusing efforts of the research community on understanding mechanisms of microbial interactions, (ii) exploring how such interactions drive persistence, antibiotic recalcitrance, and virulence, and (iii) serving as a platform to discover novel antimicrobials that are effective against such complex communities. However, one of the major hurdles in defining such a community is the existing interpatient heterogeneity in microbial community composition. For example, in one of the earliest culture-independent CF studies examining microbial communities in the lungs of pwCF, Rogers and colleagues observed that the 16S rRNA gene-based detection of key CF pathogens, such as P. aeruginosa, *Staphylococcus*, Burkholderia cepacia complex, and *Streptococcus* spp., varied from person to person (19). Subsequent 16S rRNA gene-based studies converge to the same conclusion: the microbial composition of the CF lung environment is highly variable between individuals (20–25). Similarly, in one of the first studies describing the metagenome of microbial communities from five pwCF, Lim and colleagues observed that the microbiome of each person was unique (26). They reported situations where P. aeruginosa was the most abundant microbial species in one person, but Rothia mucilaginosa and *Streptococcus* spp. were the most abundant species in another individual (26). Furthermore, while a long-term decrease in community microbial diversity over time observed in pwCF has been linked to worsened lung function (22), the impact of short-term changes in community composition in the CF airway is still a matter of debate. Some groups have reported that community structure in pwCF can shift upon drug treatment or during an exacerbation event, but those changes are transient and the communities generally remain stable over time in an individual (22, 27, 28). However, other studies have identified shifts in microbial community structure at the onset of exacerbation events (29, 30). Thus, we posit that reliable recording of and careful attention to these short- and long-term changes in the infection communities will be critical when assessing sputum samples from pwCF in cross-sectional analyses. Unless these community features are rigorously defined, associated with sputum samples, and taken into account in any investigation, the resulting analyses will likely be of limited value.

How then, can one identify and select a model community to study in the context of polymicrobial infections existing in the CF airway? With the multiple lines of evidence pointing at the heterogeneous microbial communities existing between pwCF, we would argue that this is the wrong question to ask. Instead, one should ask: are there a limited number of communities that would encompass most of the microbial diversity encountered in the CF airway? In an attempt to address this open question, we present two different analyses below.

**FIG 1:**
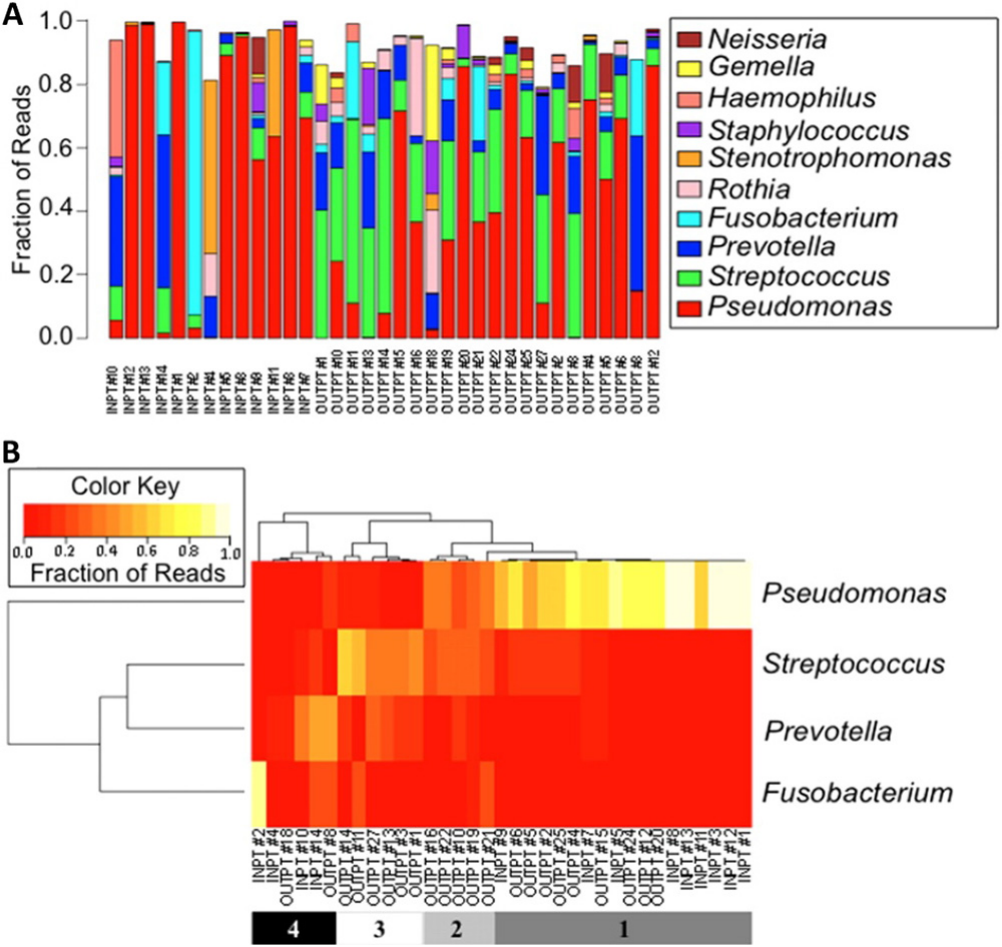
Characterization of the polymicrobial communities of sputum samples from cystic fibrosis inpatients and outpatients. (A) Fraction of 454 pyrosequencing reads assigned to each of the top 10 genera detected in the sample set as a whole is shown for sputum samples analyzed from inpatients (INPT; *n *= 13) and outpatients (OUTPT; *n *= 22) from a cross-sectional study. The legend on the right indicates the color assigned to each indicated genus. (B) Heat map of samples from pwCF based on Pearson hierarchical clustering of relative bacterial abundance using the data in panel A for the most prevalent four genera, which account for ∼86% of the total pyrosequencing reads. The legend at the bottom indicates the four clusters or “groups” assigned by hierarchical clustering, as reported (20). The airway infection communities from pwCF can be described by one of three profiles: (i) high *Pseudomonas* (group 1), (ii) high *Streptococcus* (group 3), and (iii) mixed communities with a relatively even distribution of taxa (groups 2 and 4). Modified from reference 20.

First, previous work from our team addressed this question using 16S rRNA gene data from a small number of pwCF. We analyzed sputum samples from 35 pwCF, either undergoing a clinical flaring of disease (i.e., an exacerbation) that required hospitalization (Fig. 1A, inpatients [INPT]) or pwCF who were clinically stable and attending a regular clinical visit (Fig. 1A, outpatients [OUTPT]). At first look, it is difficult to discern any patterns from the relative abundance data determined by 16S rRNA gene sequencing.

We then clustered these data based on the abundance of the four major taxa that comprised 86% of the 16S rRNA gene reads. Interestingly, clustering these data revealed four groups: *Pseudomonas*-dominated (Fig. 1B, group 1) and *Streptococcus*-dominated (Fig. 1B, group 3) communities, plus a group wherein *Pseudomonas* and *Streptococcus* were in relatively equal levels (Fig. 1B, group 2) and a group where either *Prevotella* and *Fusobacterium* were dominant (Fig. 1B, group 4), but these latter two clusters showed relatively even distribution of taxa (Fig. 1B, groups 2 and 4). Thus, three communities could explain much of the data from these 35 pwCF: *Pseudomonas*-dominated, *Streptococcus*-dominated, and evenly mixed.

**FIG 2:**
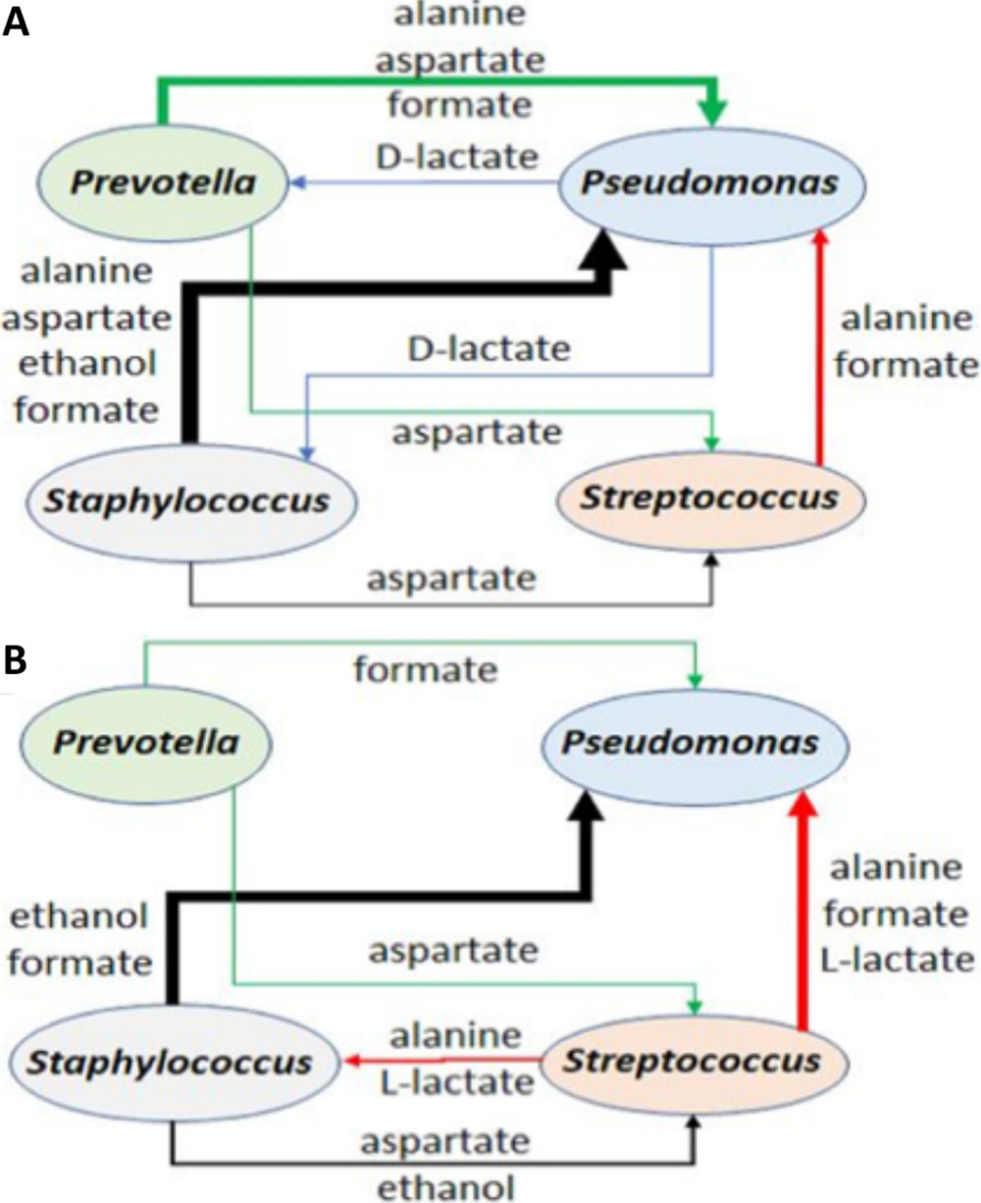
Predicted metabolite cross-feeding relationships between the four most abundant genera. One thousand model simulations were performed and split into 500 cases with relatively high *Pseudomonas* abundances and 500 cases with relatively low *Pseudomonas* abundances. The arrow thickness represents the magnitude of the metabolic exchange rate between the microbial species of the consortia. The color of the arrows is defined by the species producing and cross-feeding the metabolite(s) as follows: black arrow, *Staphylococcus*; red arrow, *Streptococcus*; blue arrow, *Pseudomonas*; green arrow, *Prevotella*. (A) Schematic representation of predicted metabolite exchange for *Pseudomonas*-dominated communities. (B) Schematic representation of predicted metabolite exchange for *Streptococcus*-dominated communities. Modified from reference 36.

Second, while 16S rRNA gene-based and metagenomic studies can allow one to determine “who is there” and probe the predicted functional capacities of the communities, these approaches are not sufficient to systematically identify and quantitatively predict the metabolic interactions existing between species observed in such communities (31). Thus, we hypothesized that by leveraging the large number of readily available published 16S rRNA gene sequence data sets in conjunction with metabolic modeling, one could also identify a small set of polymicrobial communities (we will refer to these here as “metabolotypes”) that encompass most of the airway communities for these pwCF. Metabolic modeling assembles metabolic pathway information from genome-scale reconstructions of bacterial metabolism in multiple taxonomic groups in a given community, creating a metabolotype based on predicted metabolic interactions among taxa, typically at the genus level (32). That is, beginning with genomic information, a list of produced and consumed metabolites is generated based on the predicted environment—for example, artificial sputum medium (33–35) can be used as the starting condition. An interaction map of predicted shared metabolites can then be generated to interrogate predicted metabolic interaction among community members (Fig. 2); such information is not available in 16S rRNA gene data sets. Furthermore, given that multiple genera can play the same metabolic role, it is predicted that different microbial communities have similar metabolic capabilities, thereby allowing one to collapse multiple 16S rRNA gene-predicted communities into relatively few community types.

Henson and colleagues leveraged 16S rRNA gene data from 75 sputum samples obtained from 46 pwCF to develop a community metabolic model that incorporated the 17 most abundant bacterial taxa in these samples (36). They showed that sample-by-sample heterogeneity both within patients and across patients could be captured through random variation of the simulated metabolic environment. For example, the model predicted high abundances of the “rare” pathogens *Enterobacteriaceae*, *Burkholderia*, and *Achromobacter* in three patients whose polymicrobial infections were dominated by one of these pathogens. Furthermore, this model was capable of predicting that for 43/46 pwCF analyzed, the CF sputum communities would be dominated by *Pseudomonas* (Fig. 2A), *Streptococcus* (Fig. 2B), or communities with a more even distribution of taxa (i.e., the mixed communities mentioned above), thereby providing a predictive metabolic basis for the three clusters identified from abundance data (Fig. 1). Importantly, the model yielded insights into the metabolic interactions that could support the observed metabolotypes which could not be deduced from the 16S rRNA gene data only. Model predictions indicated that the three metabolotypes were distinguished in part by the differential capacity of these organisms to metabolize amino acids, organic acids, and metabolites cross-fed by other members of the community such as *Prevotella* and *Staphylococcus*, the latter of which are found in lower abundances (36).

Thus, we suggest that metabolic modeling may be a useful approach to both collapse multiple “16S rRNA gene community types” into a smaller number of metabolotypes, and furthermore, to generate testable hypotheses regarding which specific pathways might impact community structure and function, virulence potential, and antibiotic tolerance, and ultimately, represent potential drug targets. Also, using this *in silico* approach, one has the capacity to computationally inactivate specific metabolic functions to interrogate how these pathways might impact a community (37); such flexibility in predictions will be key to understand how clinical isolates, strain variants, or mutant strains detected in pwCF might contribute to community metabolic activities in the CF lung.

Having identified community types, the next step would be to validate them experimentally by cocultivating the microbial species found in predicted metabolotypes. Validated metabolotypes would provide a small set of experimental systems one could use to understand the microbial interactions that govern clinically relevant community behavior, including antibiotic recalcitrance, ultimately generating insights that could lead to better treatments for polymicrobial infections in CF and other diseases. Although increasing model complexity can improve accuracy, we posit that modeling *in vitro* systems composed of four, five, or six of the most abundant microbes identified in metabolotypes would explain most of the interactions within a community, and therefore recapitulate most clinically relevant phenotypes. Indeed, though many groups have focused on characterizing how interactions between two bacterial species can influence various phenotypes, such as the modulation of antimicrobial efficacy or virulence, few have attempted to model community systems composed of three or more microbes (38, 39). One of the most compelling of such studies, by Vandeplassche and colleagues (40), modeled a CF-relevant community composed of six microbial species that are frequently coisolated together in the context of CF lung disease. These investigators sought to understand how antimicrobials commonly used to treat P. aeruginosa infections for pwCF (ceftazidime, tobramycin, ciprofloxacin, and colistin) could impact a polymicrobial biofilm community composed of P. aeruginosa, S. aureus, Streptococcus anginosus, Achromobacter xylosoxidans, R. mucilaginosa, and Gemella haemolysans compared to their monospecies counterparts. Interestingly, the authors observed that the antibiotic susceptibility of bacterial species grown as a community was similar to when they were grown as pure culture biofilms. This finding was surprising given previous studies showing the complex response of mixed communities to antibiotic treatment (12–15, 39, 41). Importantly, the medium used in that study was one that could sustain the growth of pure culture species biofilm (brain heart infusion broth supplemented with blood); however, this medium was unlikely to mimic conditions *in vivo*. A more representative *in vitro* system would accurately reflect the *in vivo* nutritional environment, oxygen levels, pH, etc., factors that can also be included as inputs for metabolic modeling approaches. For example, the artificial sputum medium developed by Palmer and Whiteley is thought to accurately reflect key features of the CF airway nutritional environment (33–35), and this medium can be buffered to mimic *in vivo* pH or supplemented with mucin to mirror sputum composition and viscosity.

## SUMMARY AND FUTURE DIRECTIONS

The multiple studies reporting the microbial communities in sputum and lavage fluid so far have made it clear that our hope of finding a single microbial community shared by most pwCF and responsible for driving disease is unrealistic. However, there may be a small set of communities informed by metabolic modeling, i.e., “metabolotypes” that represent the majority of CF-relevant airway infections. Identifying such microbial communities is important for several reasons. First, they provide a focus for identifying microbes needed to build *in vitro* model systems—of course such models need to strive to show growth in conditions that, to the best of our ability, reflect the *in vivo* environment. Second, such models provide key experimental systems needed to explore the mechanisms of microbial interactions that drive clinically relevant phenotypes like persistence, antimicrobial recalcitrance, and virulence factor production. Exploring these mechanisms will likely reveal novel drug targets relevant to the polymicrobial community context. Third, identifying a small number of model communities will make research results more reproducible both within and between laboratories and pave the way for the development of standard assays in the same way that specific strains of Escherichia coli or Bacillus subtilis have facilitated research in single species. Fourth, *in vitro* polymicrobial model systems can serve as screening platforms for novel therapeutics that specifically target polymicrobial infections, perhaps identifying new therapeutic classes of antimicrobials. Together, we are advocating for investigators working on a range of chronic polymicrobial infections, including CF, to leverage existing microbiome data sets, combined with bioinformatics, metabolomics data, and metabolic modeling, to build laboratory models that more closely reflect the complexity of polymicrobial infections and serve as focal points for the research efforts of the scientific community.

Much of the discussion above focused on the analysis of communities during clinical stability, and sometimes at a single time point for a given pwCF. The approaches described above might also help us understand how infection communities change over time. For example, over the lifetime of the pwCF, does the metabolotype change? Alternatively, pwCF undergo intermittent clinical flares (exacerbations), which often require hospitalization and intravenous antibiotic treatment. Thus, it might be possible to note changes in metabolotype across the disease cycle, from exacerbation to treatment to clinical recovery, versus the baseline state of those airway communities. These temporal changes in metabolotype may call for (and allow the prediction of) distinct therapeutic strategies for effective treatment of the airway infections. Indeed, a recent report examining the metabolomic profile of a large number of longitudinal sputum samples (*n* = 594) collected from six pwCF has recently been published (42). In this study, the investigators did not identify a clear microbial metabolic signature that is associated with exacerbation events across all patients. Such findings could potentially be explained by the selection of a cohort with poor lung function and/or at different stages of CF lung disease. However, we propose that more studies employing metabolomic approaches in combination with metabolic modeling will be necessary to generate a better understanding of the dynamic metabolic environment existing in the CF lung across multiple patients and across time, which might help in pinpointing key metabolic features associated with negative clinical outcomes.

Last, an important caveat to most of the microbiome studies published so far is the strong focus on bacteria; most reports have overlooked the presence of fungi and viruses and their impact on CF airway disease (26, 43, 44). Taken together, these multiple lines of evidence now make it clear that chronic CF lung infections are not restricted to bacteria and that interkingdom interactions involving viruses and fungi have the potential to be major players in CF lung disease. Thus, as we go forward, fungal and viral data, in addition to classic bacterial pathogens and anaerobic bacteria, should be incorporated into CF airway community models.
